# Preliminary evaluation of pectoralis major fascia use in robotic nipple sparing mastectomy and immediate breast reconstruction with gel implant: a retrospective comparative cohort study

**DOI:** 10.1097/JS9.0000000000003364

**Published:** 2025-09-05

**Authors:** Kuo Chen, Pengwei Lu

**Affiliations:** Department of Breast surgery, the First Affiliated Hospital of Zhengzhou University, Zhengzhou, P.R. China

**Keywords:** breast reconstruction, gel implant, pectoralis major fascia, robotic surgery, latissimus dorsi muscle flap

## Abstract

**OBJECTIVE:**

To demonstrate that the feasibility of using the da Vinci robotic XI surgical system for breast reconstruction with pectoralis major fascia instead of latissimus dorsi flaps.

**METHODS:**

A retrospective analysis was conducted on the clinical data of 33 female patients with breast cancer who were treated with robotic nipple sparing mastectomy and immediate breast reconstruction with gel implant (RNSMIBR) between September 2022 and June 2024 and met the selection criteria. The surgical techniques employed included the use of a latissimus dorsi muscle flap (LDMF) in seven cases (Group A), a LDMF without skin island in nine cases (Group B) and a pectoralis major fascia in 17 cases (Group C). The following data recorded: age, indication, body mass index, pathological type, treatment plan, operative time, complications, and postoperative aesthetic results.

**RESULTS:**

The time for unilateral RNSMIBR was (288.57 ± 108.68) minutes in group A, (272.22 ± 39.38) minutes in group B, and (88.53 ± 14.93) minutes in group C. In Group C, three patients erythematous flaps, and three patients experienced implant loss due to infection. The remaining patients expressed satisfaction with the aesthetic outcome of the procedure.

**CONCLUSIONS:**

The utilization of the pectoralis major fascia in lieu of the latissimus dorsi flap for RNSMIBR results in superior aesthetic outcomes and circumvents the potential injury to muscle tissue. Nevertheless, further investigation is required to ascertain the safety of this approach.

## Introduction

Breast cancer represents the most prevalent malignant tumor and the leading cause of cancer-related mortality among women globally, underscoring its significant impact on women’s health and well-being. The ongoing advancement of medical technology has led to the emergence of robotic surgical systems in the field of surgery. Due to its high degree of precision, flexibility, and minimally invasive nature, robotic surgical systems present a novel option for the surgical treatment of breast cancer. Since the initial report of robot-assisted surgery in 1985, its application in the context of breast cancer surgery has expanded to encompass a range of procedures, including breast-sparing surgery, breast reconstruction, flap grafting, and lymph node dissection, among others^[1–5]^. In 2015, Toesca et al[6]. reported the first instance of robotic nipple sparing mastectomy (RNSM). Since then, this technique has been gradually applied and developed worldwide, demonstrating promising prospects for future applications. Please see surgical video (http://links.lww.com/JS9/F15).

**HIGHLIGHTS**

- **Innovative Application of the Pectoralis Major Fascia in Robotic Breast Reconstruction (RNSMIBR):** This study is the first to utilize the pectoralis major fascia as a substitute for the traditional latissimus dorsi musculocutaneous flap (LDMF) in RNSMIBR. This technique offers structural support for the reconstructed breast, achieves favorable aesthetic outcomes, significantly shortens the operative time, and reduces surgical trauma, showing potential for wider clinical adoption.
- **Reconstruction Strategy that Balances Aesthetics and Muscle Function Preservation:** Compared with conventional LDMF techniques, this fascia-based approach avoids damage to the back musculature, preserving physical function and dorsal aesthetics. It is particularly suitable for patients who are not ideal candidates for back flap harvesting.
- **Comprehensive Comparison of Two Robotic Breast Reconstruction Techniques:** The study systematically reviews current advances and clinical outcomes of robotic-assisted LDMF harvesting and highlights its limitations, such as extended operative time and a steep learning curve. It also preliminarily assesses the feasibility and complications of fascia-based reconstruction, providing a theoretical foundation for surgical optimization.
- **Clinical Alert for Post-Radiotherapy Patients:** The pectoralis major fascia demonstrates lower radiotherapy tolerance, with a higher risk of complications such as fascia necrosis and implant exposure, especially in patients undergoing postoperative radiotherapy. The study accordingly recommends cautious patient selection and advises against using this approach in radiotherapy candidates.
- **Promotion of Standardized Pathways in Robotic Breast Reconstruction:** This research reveals the current lack of standardized procedures in robotic breast reconstruction and emphasizes the need for further refinement in surgical protocols, training systems, and patient selection criteria.

In the context of breast cancer surgery, breast reconstruction constitutes an essential element in the enhancement of patients’ quality of life. The use of a LDMF for breast reconstruction results in a more natural shape than that achieved with implants and is more tolerant of radiotherapy. The success of the surgical procedure is contingent upon the successful acquisition of the flap^[7,8]^. With the advent of robotic surgical systems, the role of these systems in flap transplantation has been the subject of extensive study. Nevertheless, in patients with substantial breast volume and excessive ptosis, an optimal aesthetic outcome remains unattainable. Furthermore, the acquisition of the LDMF results in some degree of trauma to the back muscle tissue, which subsequently restricts the patient’s ability to engage in physical activities that require movement of the back. The purpose of this retrospective study was to demonstrate that the feasibility of using the da Vinci robotic XI surgical system for breast reconstruction with pectoralis major fascia instead of LDMF.

To our knowledge, this is the first clinical study to explore the feasibility and outcomes of using the pectoralis major fascia, rather than the traditional LDMF, for immediate breast reconstruction following RNSMIBR. While robotic-assisted flap techniques have been reported in prior studies, the use of the pectoralis major fascia as an autologous support structure in this context has not been previously evaluated in the literature. This novel approach aims to reduce donor-site morbidity, simplify surgical access, and shorten operative time, while preserving satisfactory aesthetic outcomes. By presenting this new technique and analyzing its early outcomes, our study contributes to expanding the surgical repertoire for robotic breast reconstruction and offers a potential alternative for selected patient populations. This retrospective comparative cohort study has been reported in line with the STROCSS guidelines[9].

## Materials and methods

### Patients and criteria

The study population comprised 33 female patients with breast cancer who were treated with RNSMIBR at the Department of Breast Surgery of the First Affiliated Hospital of Zhengzhou University between September 2022 and June 2024. Of these patients, 10 had undergone contralateral breast mammaplasty (CBM). In order to be eligible for inclusion in the study, patients had to meet the following criteria: (1) They had to be female patients diagnosed with unilateral breast cancer. (2) Patients who were not eligible for breast conserving surgery according to the National Comprehensive Cancer Network (NCCN) guidelines. (3) Sagging of the breast morphology. (4) Patients who are willing to undergo RNSMIBR surgical treatment. The following criteria were used to exclude patients from the study: (1) Allergy to anesthetic agents; (2) Mental illness and combination of severe cardiac, pulmonary, hepatic, and renal insufficiency and other contraindications to surgery; (3) History of drug abuse. The surgical techniques employed included the use of a latissimus dorsi muscle flap (LDMF) in 7 cases (Group A), a LDMF without skin island in 9 cases (Group B), and a pectoralis major fascia in 17 cases (Group C).

All patients included in the study presented with varying degrees of breast ptosis and were not suitable candidates for breast-conserving surgery. The grouping was based on preoperative evaluation of breast volume, degree of ptosis, tumor characteristics, and skin condition. Specifically, patients in Groups A and B had large, severely ptotic breasts, with an estimated glandular excision volume exceeding 500 g. Group A was selected when tumor invasion of the overlying skin or poor skin quality necessitated skin replacement. Group B included patients with similar breast volume and ptosis but with intact skin envelopes, making skin replacement unnecessary. Thus, the primary difference between Groups A and B was the need for skin coverage rather than volume replacement. Group C consisted of patients with moderately ptotic breasts and relatively smaller glandular volumes, typically suitable for prosthesis placement ≤375 cc, and with preserved skin integrity. All surgical plans were tailored based on a multidisciplinary assessment of anatomical and oncologic considerations. Although this retrospective single-center study carries an inherent risk of selection bias, no significant baseline differences were observed across groups (Table 1). A prospective, multicenter study is currently being planned to validate these selection criteria and improve external applicability.

**Table 1:** Comparison of baseline data between the 3 groups.

Baseline data	Age (x ± s, year)	BMI (x ± s, kg/m^2^)	Pathological type [*n* (%)]		Contralateral CBM surgery [*n* (%)]			Axillary lymph node surgery [*n* (%)]		T stage [*n* (%)]					N stage [n(%)]				ER [n(%)]		PR [n(%)]		HER-2 [n(%)]	
			IDC	DCIS	No		Yes	SLNB	SLNB-ALND/ALND	TIS	T1	T2	T3	T4	N0	N1	N2	N3	Positive	Negative	Positive	Negative	Overexpression	Negative
Group A *n* = 7	37.29 ± 4.27	23.47.1 ± 0.99	7 (100)	-	2 (28.6)		5 (71.4)	2 (28.6)	5 (71.4)	-	2 (28.6)	2 (28.6)	2 (28.6)	1 (14.3)	2 (28.6)	3 (42.8)	2 (28.6)	-	4 (57.1)	3(42.9)	4 (57.1)	3 (42.9)	2 (28.5)	5 (71.5)
Group B *n* = 9	39.11 ± 5.74	24.67 ± 2.58	8 (88.9)	1 (1.1)	3 (33.3)		6 (66.7)	4 (44.4)	5 (55.6)	1 (1.1)	4 (44.4)	4 (44.4)	-	-	5 (55.5)	1 (11.1)	3 (33.3)	-	7 (77.8)	2 (22.2)	9 (100)	-	2 (22.2)	7 (77.8)
Group C *n* = 17	46.24 ± 6.98	25.09 ± 2.06	16 (94.1)	1 (5.9)	16 (94.1)		1 (5.9)	10 (58.8)	7 (41.2)	1 (5.9)	4 (23.5)	11 (64.7)	1 (5.9)	-	10(58.8)	2 (11.8)	4 (23.5)	1 (5.9)	10 (58.8)	7 (41.2)	9 (52.9)	8 (47.1)	6 (35.3)	11 (64.7)
Statistical value	F = 6.349	F = 1.409	χ2 = 0.856		χ2 = 10.969			χ2 = 10.764		χ2 = 9.530					χ2 = 5.051				χ2 = 1.042		χ2 = 6.227		χ2 = 0.489	
*P*-value	0.005**	0.260	0.652		0.004**			0.005**		0.146					0.537				0.594		0.044*		0.783	
**Baseline data**		**AC** [*n* (%)]				**NAC** [*n* (%)]				**Radiotherapy** [*n* (%)]					**Breast sagging** [*n* (%)]									
															0				I				II	
Group A *n* = 7		7 (100)				5 (71.4)				5 (71.4)					2 (28.6)				2 (28.6)				3 (42.8)	
Group B *n* = 9		8 (88.9)				4 (44.4)				4 (44.4)					-				4 (44.4)				5 (55.5)	
Group C n = 17		16 (94.1)				2 (11.8)				7 (41.2)					-				5 (29.4)				12 (70.9)	
Statistical value		χ2 = 0.856				χ2 = 8.630				χ2 = 1.898					χ2 = 8.569									
*P*-value		0.652				0.013*				0.387					0.073									

* *P* < 0.05 **P < 0.01. DCIS: Ductal carcinoma in situ. IDC: Invasive ductal carcinoma. CBM: Contralateral breast mammaplasty.

**AC**: Adjuvant chemotherapy. **ER**: Estrogen receptor. **HER-2**: Human epithelial growth factor receptor type 2. **NAC**: Neoadjuvant chemotherapy. **PR**: Progesterone receptor.

The surgical procedures were conducted with the da Vinci XI robotic surgical system (Intuitive Surgical Corp., Sunnyvale, CA, USA), and all patients received Mentor™ anatomical Gel implants (Mentor Worldwide LLC, USA). The AirSeal® system was employed to maintain a constant pressure of 12 mmHg (1 mmHg = 0.133 kPa) throughout the procedure. Prior to their participation in the study, patients were required to provide informed consent.

All surgical procedures were performed by the same lead breast surgeon, who had substantial experience with the Da Vinci robotic system and had completed the associated learning curve prior to the commencement of this study. The surgeon received comprehensive robotic surgery training at the Intuitive Surgical Training Center in Shanghai, China, including simulator-based instruction and hands-on animal model procedures. This standardized training ensured the surgeon’s proficiency in robotic techniques and qualification for clinical application, thereby minimizing operator-related variability and reducing potential biases associated with the learning curve.

### Surgical techniques

#### RNSM

**Figure 1.**
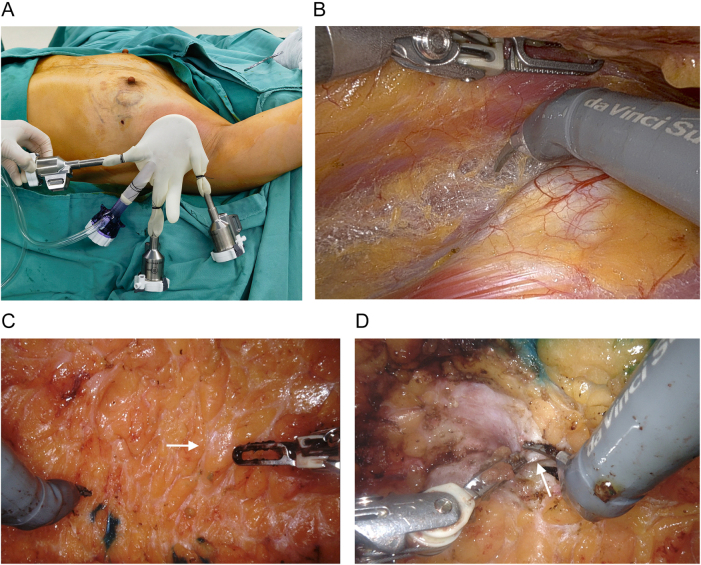
(A) After sentinel lymph node biopsy, installation of a single-hole protective sleeve at the incision, carbon dioxide (CO_2_) was inflated with air pressure kept at 12 mmHg to create space for mastectomy. (B) Placement of robotic arms and the robotic arm passing around the rib cage with a large degree of flexion to disconnect the posterior pectoralis major gap. (C) The Scarpa fascia plane is visible on the surface of the gland (white arrow). (D) A robotic arm is employed to perform a biopsy of the posterior cut edge of the nipple (white arrow).

The RNSMIBR procedure is conducted under general anesthesia. The patient was positioned in the supine position with shoulder pads placed beneath the scapula and the shoulder joint and upper extremity abducted. Subsequently, a 4–5 cm incision was made at the junction of the midline of the axilla and the upper edge of the breast on the affected side. The cavity was constructed from the incision made in the axilla, exposing the outer edge of the pectoralis minor muscle, the pectoralis major interosseous muscle, the surface of the pectoralis major muscle, and Scarpa’s fascia. A cavity measuring approximately 5 cm by 5 cm was created. A single-hole protective sleeve was then installed at the incision site (Fig. 1A). A methylene blue stain was injected at the edges of the gland in the direction of the 3, 6, 9, and 12 points in order to facilitate intraoperative positioning. The pectoralis major and pectoralis minor interspace is initially released, and the pectoralis major muscle is truncated at the lower crease (Fig. 1B). Subsequently, the mammary gland is released from its attachment to the pectoralis major muscle, extending to the edge of the gland. Subsequently, the robotic instrument was operated to access the Scarpa fascia plane on the surface of the gland, and the gland was resected along this plane, ensuring the preservation of the subcutaneous fat layer (Fig. 1C). A biopsy was conducted on the posterior nipple margin (Fig. 1D).

**Figure 2.**
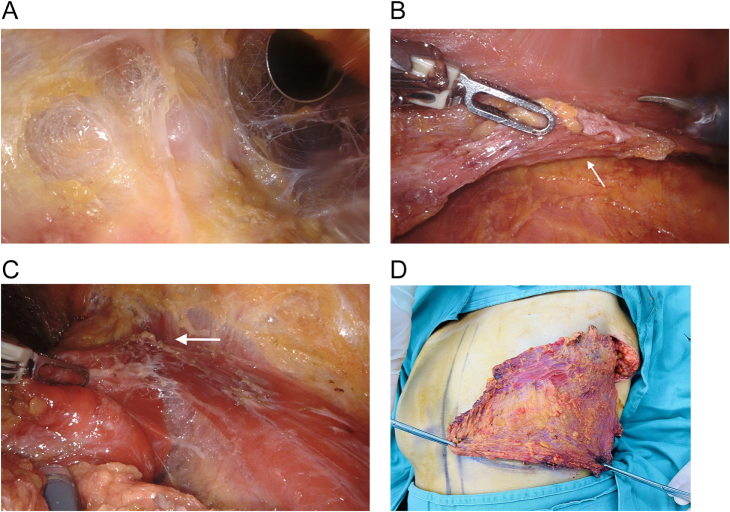
(A) Surgical field formed after lipolysis. (B) The LDMF was peeled using a robot (white arrow). (C) It is of the utmost importance to ensure the protection of the vascular tip section of the LDMF (white arrow). (D) The LDMF without skin island was repositioned to the chest, and the patient’s position was adjusted accordingly.

#### Acquisition of the LDMF

A lipolytic solution was prepared and ultrasound-guided injections were administered into the subcutaneous adipose tissue in the vicinity of the anterior border of the LDMF. Subsequently, liposuction was conducted to create a cavity in the region of the anterior border of the LDMF (Fig. 2A). The subcutaneous tissue along the axillary incision should be released to the anterior border of the LDMF. The robotic instrument arm should then be used to release the tissue LDMF, gradually preserving the subcutaneous fat, and freeing the LDMF from the midline outward (Fig. 2B). It is essential to safeguard the dorsal thoracic artery on the side adjacent to the lesser tuberosity of the humerus. Additionally, the excess tissue at the tip of the LDMF should be excised to enable the flap to be rotated (Fig. 2C). At this juncture, the dorsal and axillary cavities are connected, and the LDMF is rotated to the anterior end of the chest via this conduit (Fig. 2D).

#### Acquisition of the pectoralis major fascia

**Figure 3.**
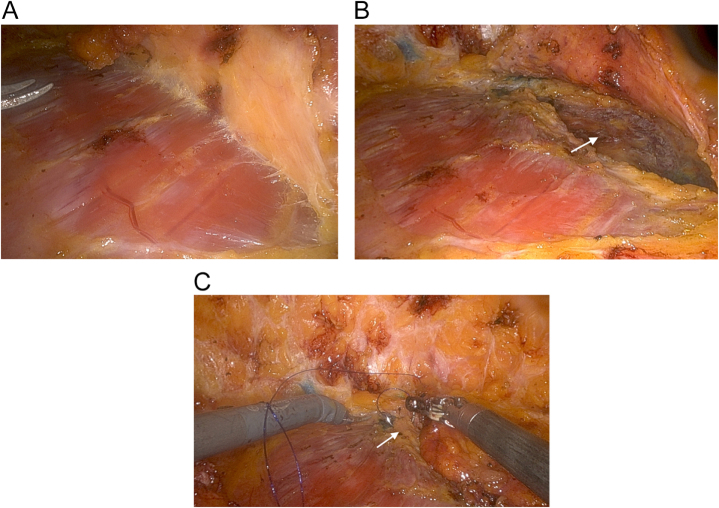
(A) The pectoralis major fascia is meticulously peeled using a robotic arm in order to prevent rupture. (B) The inferior border of the pectoralis major muscle is visible within the surgical field, having been truncated (white arrow). (C) the acquired pectoralis major fascia is sutured to the truncated the pectoralis major muscle (white arrow).

**Figure 4.**
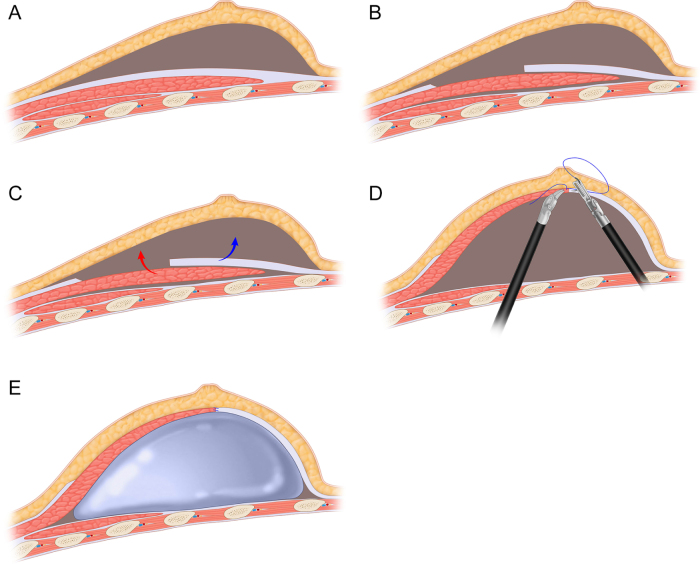
(A, B, C, D, E) The expansion of the longitudinal space of the posterior pectoral “pouch”.

The pectoralis major fascia is meticulously stripped in the plane of the nipple toward the foot with the aid of a robotic manipulator arm (Fig. 3A). The dexterous capabilities of the robot facilitate the complete stripped of the pectoralis major fascia from the surface of the pectoralis major muscle in a single piece. The fascia was incised to the level of the inframammary wall, at which point the truncation of the lower edge of the pectoralis major muscle was visible within the surgical field (Fig. 3B). Subsequently, the superior edge of the acquired pectoralis major fascia is sutured to the truncated lower edge of the pectoralis major muscle (Fig. 3C). This results in an expansion of the longitudinal space of the posterior pectoralis major muscle “pouch.” The recently formed “pouch” structure may result in a breast reconstruction that appears to be more ptotic (Fig. 4).

#### Intraoperative techniques for skin flap protection

Breast mastectomy was performed using the robotic surgical system, employing bipolar grasping forceps and monopolar electroshears. The settings for bipolar and monopolar electrocoagulation were 45 W and 30 W, respectively. To optimize flap vascularity and minimize postoperative complications, robotic instruments were used to navigate along the Scarpa fascia plane in order to preserve a thick subcutaneous fat layer (Fig. 1C). However, at the tumor site, the dissection plane was deliberately shifted to the Camper fascia layer to ensure oncologic safety and achieve complete tumor excision.

**Figure 5.**
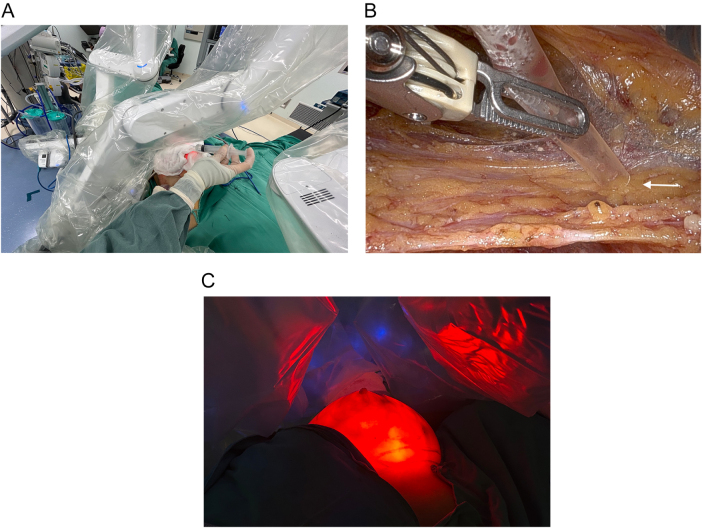
(A) Intraoperative coverage of the breast surface with ice-water-soaked gauze. (B) The suction device is temporarily fixed to surrounding fat using negative pressure, helping maintain the workspace and allowing repositioning during dissection(white arrow). (C) The extent and thinness of the excision can be determined by visual inspection of the light source after the removal of the gland.

To minimize thermal injury to the skin flap, sterile gauze soaked in ice water was applied to the breast surface throughout the procedure (Fig. 5A). Additionally, during gland resection, ice-cooled gauze was used to shield the skin from heat steam generated by electrocautery, thereby reducing the risk of thermal damage. A custom-made smoke and heat steam evacuation system was constructed to further protect the skin and improve intraoperative visibility. After the breast surface was covered with gauze, a suction device was assembled using a transfusion hose, an inflatable interface, and an external suction source. This device could be manipulated with robotic grasping forceps to effectively remove smoke and hot vapor during the procedure. However, continuous suction led to a reduction in CO₂ pressure, which could compromise the surgical workspace. To address this, the suction apparatus was temporarily affixed to the adipose tissue via negative pressure and repositioned as needed during dissection (Fig. 5B). To ensure a stable operative field, the AirSeal® system was implemented, which helped maintain a consistent CO₂ pressure within the breast cavity. Following resection, the robotic light source allowed for real-time assessment of flap uniformity and thickness through the translucent skin, confirming adequate gland removal and flap viability (Fig. 5C).

#### Indicators are used to assess operative time

To assess the impact of accumulated case experience on RNSMIBR operative time, the operative time required for unilateral RNSMIBR surgery was quantified. The total operative time was affected by the axillary lymph node approach, CDM of the contralateral breast, and the need to change the patient’s position to obtain the latissimus dorsi flap, making it impossible to accurately compare the total operative time among the three groups. The term “operative time” refers to the total time from the installation of the robotic surgical platform to the start of the surgical instruments to the removal of the surgical instruments. This includes the time to perform the breast mastectomy, harvest the LDMF, and acquisition of pectoralis major fascia.

#### Statistical analyses

All statistical analyses were conducted using SPSS version 26.0. Continuous variables were first tested for normality using the Kolmogorov–Smirnov test. Data conforming to a normal distribution were expressed as mean ± standard deviation (SD) and compared using one-way analysis of variance (ANOVA); effect sizes were estimated using Cohen’s f with 95% confidence intervals. Non-normally distributed data were presented as median (interquartile range) and compared using non-parametric tests. Categorical variables were expressed as counts and percentages [*n* (%)] and analyzed using the Chi-square test (χ^2^), with effect size assessed by Cramér’s V. Ranked data were compared using the rank sum test. A two-tailed *P*-value of < 0.05 was considered statistically significant. No adjustments for potential confounders were made in this analysis.

#### Evaluation of surgical results

To comprehensively assess surgical safety and efficacy, both objective clinical indicators and subjective patient-reported outcomes were evaluated in this study. Postoperative complications were graded using the Clavien–Dindo classification system, a widely adopted and objective method in surgical practice for evaluating the severity of complications. This system categorizes complications into five grades based on the required level of intervention and impact on patient health: Grade I (minor complications not requiring specific treatment), Grade II (requiring pharmacological treatment), Grade III (requiring surgical, endoscopic, or radiological intervention), Grade IV (life-threatening complications requiring ICU care), and Grade V (death). The grading system offers a concise and standardized framework for comparing perioperative safety across patient groups and surgical techniques. Patient-reported aesthetic and quality-of-life outcomes were assessed using the BREAST-Q questionnaire, a validated, procedure-specific instrument designed for individuals undergoing breast surgery, including reconstruction, reduction, and augmentation. The BREAST-Q encompasses multiple dimensions, such as satisfaction with breast appearance, psychosocial well-being, physical well-being, sexual well-being, and postoperative discomfort. Its strong psychometric properties and international applicability make it a reliable tool for measuring patient-centered outcomes in breast surgery research. In this study, patient satisfaction scores derived from the BREAST-Q were categorized into four levels – Excellent, Good, Moderate, and Poor – based on standardized scoring criteria.

## Results

**Figure 6.**
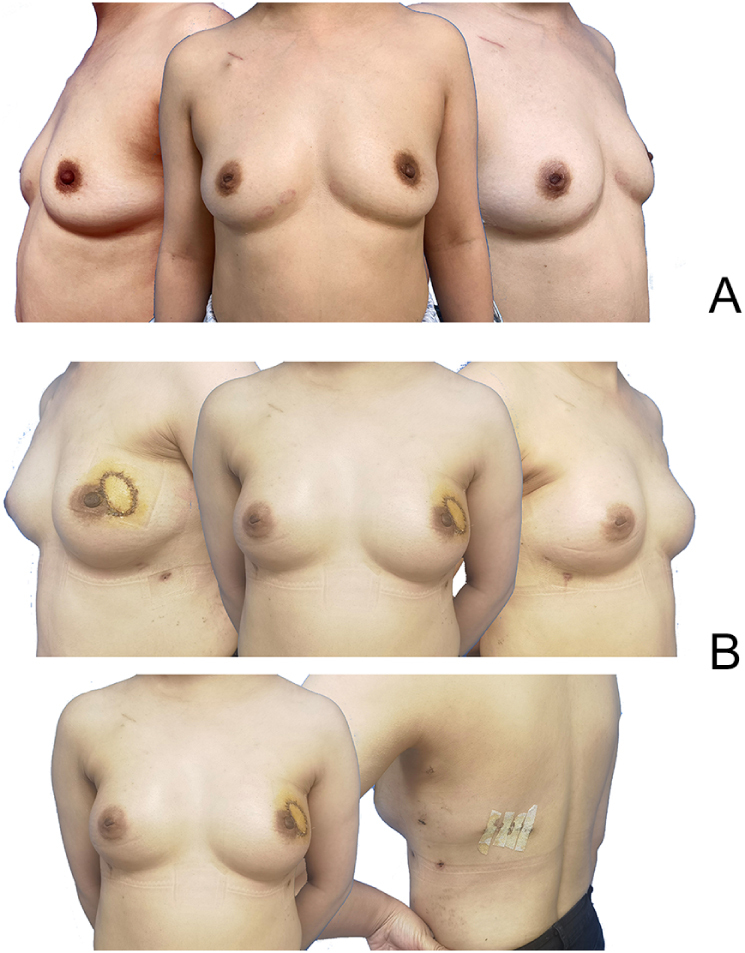
Comparison of preoperative and postoperative outcomes in patients undergoing RNMSIBR-LDMF in the left breast, CBM performed on the right breast. (A) Preoperative. (B) Three weeks postoperative.

**Figure 7.**
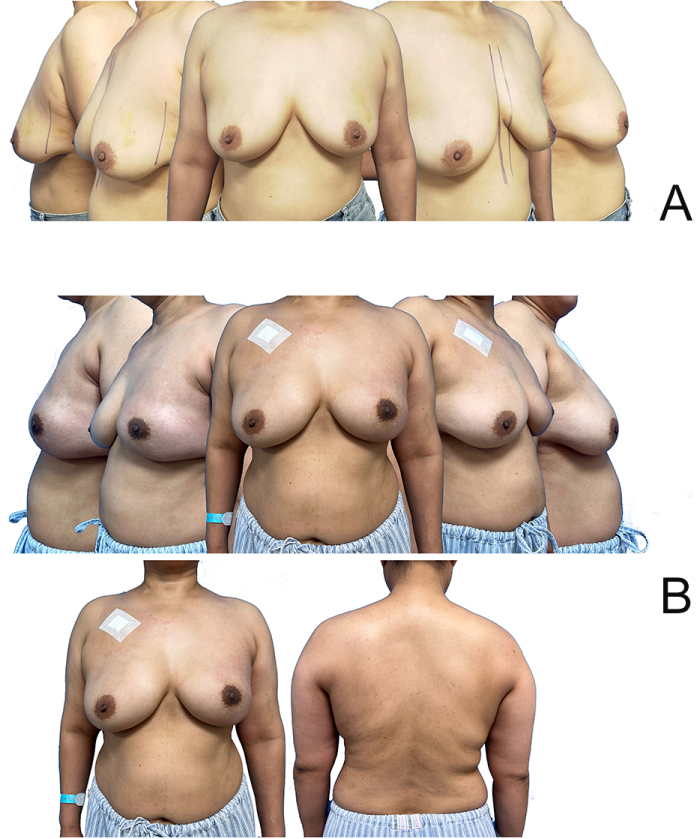
Comparison of preoperative and postoperative outcomes in patients undergoing RNMSIBR-LDMF without skin island in the left breast. (A) Preoperative. (B) Three weeks postoperative.

**Figure 8.**
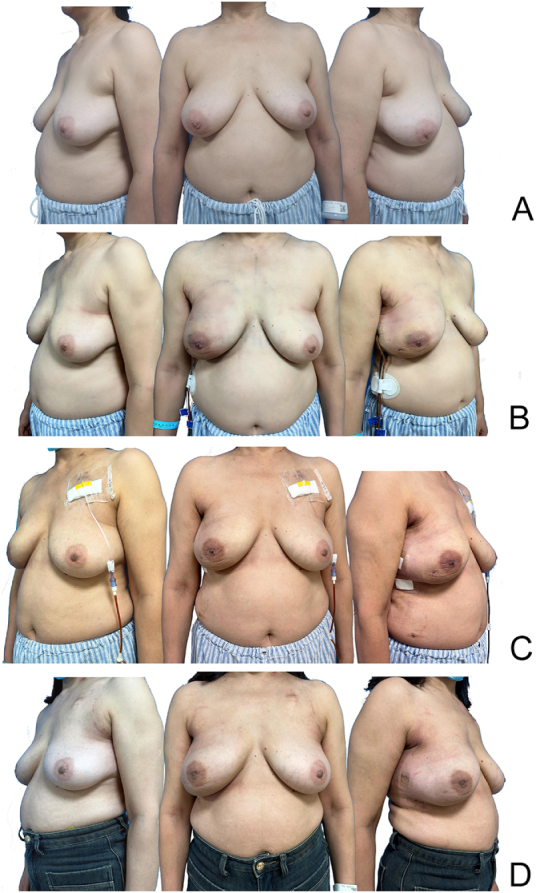
Comparison of preoperative and postoperative outcomes in patients undergoing RNMSIBR – pectoralis major fascia in the right breast. (A) Preoperative. (B) Two days postoperative. (C) Two weeks postoperative. (D) Three months postoperative.

The RNSMIBR surgical procedure was successfully completed, and there were no instances of conversion to open surgery. No recurrence of metastasis was observed in any of the three groups during the postoperative follow-up period of 10 months, with a standard deviation of one month. The mean operative times for the three groups were as follows: Group A: 288.57 ± 108.68 minutes, Group B: 272.22 ± 39.38 minutes, and Group C: 88.53 ± 14.93 minutes (*P* = 000). The positive rate of the posterior nipple margin was 0, and there were no complications, such as necrosis of the nipple-areola complex, in the three groups. One patient in Group C experienced a loss of the implant due to a retrograde infection that occurred at home following discharge from the facility. This was caused by the absence of an aseptic dressing change of the drain opening. In one patient, infection resulted in flap necrosis, implant exposure, and implant loss. In another patient, radiotherapy caused flap necrosis, implant exposure, and ultimately, implant loss. Three patients in Group C exhibited erythematous flap. Postoperative aesthetic outcomes were evaluated using the BREAST-Q questionnaire, BREAST-Q assessments were completed for all three groups. The majority of patients reported high levels of satisfaction, with responses concentrated in the “Excellent” and “Well” categories. Notably, patients who rated the outcome as “Poor” was also the one who experienced implant loss, suggesting a possible link between postoperative complications and reduced aesthetic satisfaction. (Figures 6–8) (Table 2).

**Table 2:** Comparative analysis of surgical procedures and outcomes in three groups.

Indicators	Group A (*n* = 7)	Group B (*n* = 9)	Group C (*n* = 17)	Statistical test	*P*-value	Effect size (95% CI)
Operation time (min, mean ± SD)	288.57 ± 108.68	272.22 ± 39.38	88.53 ± 14.93	ANOVA (F = 44.995)	0.000**	**Cohen’s *f* = 1.56** (1.09–2.00)
Hemorrhage (ml, mean ± SD)	31.43 ± 8.75	25.00 ± 5.77	10.59 ± 1.61	ANOVA (F = 44.464)	0.000**	**Cohen’s *f* = 1.50** (1.05–1.95)
Postoperative complications (Clavien–Dindo)	0	0	II-5(29.4%)			
			III-3(17.6%)			
Erythematous flap [*n* (%)]	0 (0%)	0 (0%)	3 (17.6%)	Chi-square	0.129	Cramér’s V = 0.37 (0.00–0.70)
Flap necrosis [*n* (%)]	0 (0%)	0 (0%)	2 (11.8%)	Chi-square	0.249	Cramér’s V = 0.29 (0.00–0.62)
Loss of implant [*n* (%)]	0 (0%)	0 (0%)	3 (17.6%)	Chi-square	0.129	Cramér’s V = 0.37 (0.00–0.70)
Breast-Q Aesthetic Outcome						
Excellent	6 (85.7%)	7 (77.8%)	14 (82.4%)			
Well	1 (14.3%)	1 (11.1%)	0 (0%)			
Moderate	0 (0%)	1 (11.1%)	0 (0%)			
Poor	0 (0%)	0 (0%)	3 (17.6%)	Chi-square = 5.873	0.438	Cramér’s V = 0.41 (0.05–0.73)

* *P* < 0.05 **P < 0.01.

## Discussion

Breast reconstruction surgery has the potential to alleviate post-surgical complications and improve the overall quality of life for breast cancer patients. RNSMIBR has been shown to be a feasible and generally safe procedure. A recent systematic review of 249 cases reported low intraoperative complication rates, with only two conversions due to bleeding and no perioperative deaths. Most complications, such as seroma or skin ischemia, were mild and manageable. No locoregional recurrences were observed during follow-up, supporting the short-term oncological safety of the procedure^[10–12]^. Autologous latissimus dorsi flap harvesting is a common technique used for breast reconstruction. However, this approach has the drawback of damaging the back musculature. In this study, we present the use of pectoralis major fascia for RNSMIBR, which allows for the creation of a breast with a similar appearance to that of the original breast. We also discuss the early experiences with this robotic surgery, where patients may have excellent postoperative aesthetic results, but still have a high complication rate.

The latissimus dorsi flap is one of the most commonly utilized autologous flaps in clinical practice for breast reconstruction, provided that it has an adequate blood supply and an appropriate tissue volume. The conventional open surgical excision of the latissimus dorsi muscle typically results in the formation of a 15–30 cm long surgical scar in the dorsal donor region, which can adversely impact the physiological curvature and aesthetic appeal of the back. In 2012, Selber et al[13]. published the inaugural feasibility study of robotic acquisition of latissimus dorsi muscle specimens on cadavers, accompanied by a clinical follow-up study of five cases of breast reconstruction with acquired latissimus dorsi flaps. During the course of the study, the time required for flap acquisition by the robotic system was reduced from a period of more than 2 hours to approximately 1 hour, and there were no instances of donor-site hematomas. In a case report published in 2018, Lai et al[14]. described a patient who underwent a single axillary incision robot-assisted resection of breast cancer and robotic latissimus dorsi flap harvesting for immediate breast reconstruction. The procedure was completed in a total of 97 minutes. The feasibility of the method was demonstrated by the absence of local recurrence, distant metastasis, or mortality during the subsequent 5-month follow-up period. Fouarge et al[15]. conducted a retrospective analysis of clinical data from six patients who had undergone robot-assisted latissimus dorsi flap surgery. The mean time for flap removal was 110 minutes, with a reduction in procedure time observed with increasing surgeon proficiency. Additionally, none of the six patients experienced postoperative complications. Furthermore, our study demonstrates that robotic latissimus dorsi flap grafting is a safe and feasible procedure. Nevertheless, the application of this technique has some limitations in patients with large, excessively ptotic breasts and in those who rely on their back muscles for work. In patients with excessive ptosis and no skin invasion by the tumor, we perform RNSMIBR using LDMF without skin island. This surgical approach is considered to be an important one that can produce good cosmetic results. Nevertheless, the findings of our study indicate that the procedure involving LDMF may necessitate a longer surgical time frame and increased intraoperative bleeding. Related studies have demonstrated that the current challenges associated with this robotic surgical approach can be attributed to the lengthy surgical times, which are a consequence of the lack of standardized procedures for the learning curve^[16,17]^.

The pectoralis major fascia is a connective tissue membrane located on the upper side of the pectoralis major muscle. Its primary role is to encase, protect, and support the pectoralis major muscle, as well as maintain the position and shape of the breast. It provides a natural support structure for breast reconstruction. In our study, we employed a novel approach by utilizing the pectoralis major fascia in lieu of LDMF for breast reconstruction. The findings indicated that, while an optimal result can be breast sagging achieved, the approach is more accessible and the surgical duration can be significantly reduced in comparison to LDMF under robotic surgery. Nevertheless, the incidence of complications remains relatively high.

**Figure 9.**
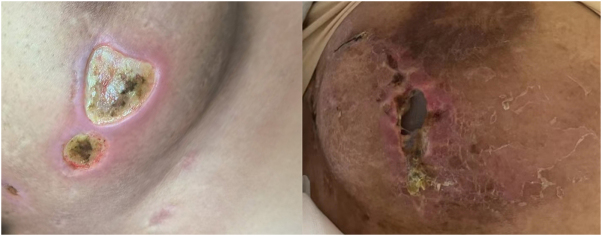
Radiotherapy induced dermatological complications ultimately result in the exposed and eventual loss of the implant, due to the necrosis of the pectoralis major fascia.

**Figure 10.**
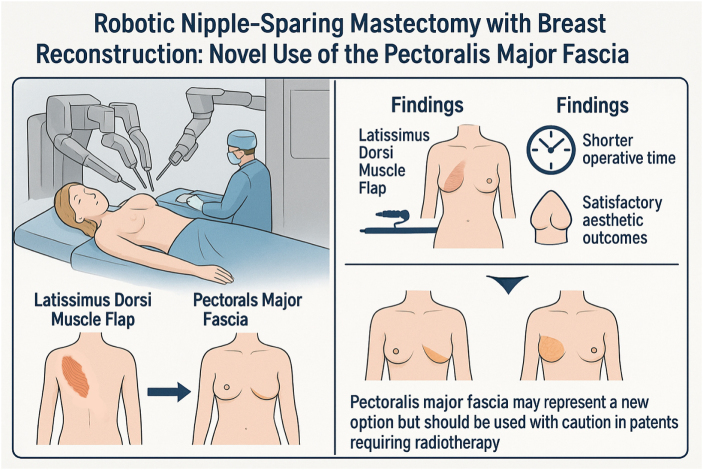
Graphical abstract.

Our study identified a relatively higher incidence of implant loss and fascia necrosis in patients undergoing breast reconstruction using the pectoralis major fascia technique, particularly among those who received postoperative radiotherapy. Several factors may contribute to these complications (Fig. 9). First, the pectoralis major fascia appears to be less tolerant to radiotherapy compared to the LDMF. In cases of radiation-induced skin injury, the fascia’s limited tissue volume and vascularity may predispose it to necrosis, ultimately resulting in implant exposure or loss. Second, surgical manipulation may compromise the fascia’s already tenuous blood supply, increasing the risk of infection and subsequent tissue breakdown. In light of these findings, we recommend that this technique be used with caution, particularly in patients with a high likelihood of requiring adjuvant radiotherapy. For such patients, LDMF reconstruction may be more suitable, as it provides a well-vascularized, robust soft tissue coverage that is more resilient to radiation-related complications. Additionally, patients with large and ptotic breasts may benefit more from LDMF-based reconstruction due to greater tissue demands. To minimize risks, a comprehensive preoperative multidisciplinary evaluation – including input from oncologists, radiologists, and reconstructive surgeons – is essential to assess the likelihood and timing of radiotherapy. If postoperative radiation is anticipated, surgeons should consider alternative flap-based methods or staged reconstruction strategies to enhance safety and long-term outcomes. Given the retrospective nature of this study, the small sample size, and limited follow-up, our findings should be interpreted cautiously. Nonetheless, based on our preliminary clinical experience, we advise against the use of pectoralis major fascia reconstruction in patients expected to undergo radiotherapy, pending further validation from larger prospective studies (Fig. 10).

This study has several limitations. As a retrospective, single-center analysis with a relatively small sample size, the findings may be subject to selection bias and limited generalizability. Differences in surgical expertise, institutional protocols, and patient populations across regions may influence the reproducibility of outcomes. To address these limitations, a prospective, multicenter study is currently being planned in collaboration with several tertiary hospitals across different regions. The aim is to further validate the safety, feasibility, and adaptability of the robotic surgical system in diverse clinical settings, and to provide more robust evidence for its broader clinical application.

## Conclusions

The utilization of the pectoralis major fascia in lieu of the latissimus dorsi flap for RNSMIBR results in superior aesthetic outcomes and circumvents the potential injury to muscle tissue. Nevertheless, further investigation is required to ascertain the safety of this approach.

## Data Availability

The data that support the findings of this study are available from the authors but restrictions apply to the availability of these data, which were used under license from the First Affiliated Hospital of Zhengzhou University for the current study, and so are not publicly available. Data are, however, available from the authors upon reasonable request and with permission from the First Affiliated Hospital of Zhengzhou University.

## Research registration unique identifying number (UIN)

This study was approved by the institutional ethics committee of the Chinese Clinical Trial Registry (ChiCTR). The study protocol was reviewed and approved by [ChiCTR] approval number [ChiCTR2500096970].
